# Impact of Low-Dose Computed Tomography on Computed Tomography Orders and Scan Length

**DOI:** 10.31486/toj.19.0008

**Published:** 2019

**Authors:** Curtis Simmons, James Milburn

**Affiliations:** ^1^ Department of Radiology, Mayo Clinic, Rochester, MN; ^2^ Department of Radiology, Ochsner Clinic Foundation, New Orleans, LA

**Keywords:** *Dose-response relationship–radiation*, *radiation exposure*, *radiology*, *retrospective studies*, *tomography–x-ray computed*

## Abstract

**Background:** New techniques have reduced the radiation dose delivered from a computed tomography (CT) examination. These techniques do not affect the number of scans ordered, the number of phases in each examination, or the scan length, as these parameters are controlled by ordering providers and CT technologists. The purpose of this study was to determine if deploying low-dose CT resulted in an increase in radiation exposure because of more liberal ordering habits or more liberal scanning ranges.

**Methods:** We identified the most frequent CT examination types through a retrospective study of billing data from 2013. A campaign for low-dose CT scans was implemented, and data from 2 months prior and 2 months after were collected (n=797; average age=51.0 years ± 20.5; range, 4 to 97 years) and analyzed for differences in radiation dose, overall area scanned, and number of phases requested using unpaired *t* tests.

**Results:** According to the billing data, the largest category of CT scans was the abdominal CT (31% of all CT examinations). After the low-dose campaign was implemented, we observed no difference in the number of examination phases ordered (1.2 ± 0.5 vs 1.3 ± 0.6, *P*=0.15), no increase in length of the scan (45.1 ± 7.5 cm vs 43.7 ± 10. 4 cm, *P*=0.08), and an overall decrease in dose (1,069 ± 634 mGy*cm vs 676 ± 480 mGy*cm, *P*<0.001).

**Conclusion:** A campaign alerting staff to the availability of low-dose CT did not cause an increase in CT examination ordering and did not impact the area scanned by technologists.

## INTRODUCTION

### Medical Radiation

Computed tomography (CT) scans have become the dominant source of radiation, especially in the United States.^1,2^ CT usage jumped from 6.1 scans per 1,000 people in 1970 to 48 scans per 1,000 people in the mid-1990s.^3^ The total number of CT scans that each patient receives has a right-skewed distribution, with 33% having >5 CTs, 5% having >22, and 1% having >38.^4^ Some studies, such as CT urography, require multiple phases that increase the dose of a single examination substantially.^5^ As physicians of any type become more concentrated geographically and more specialized, the absolute number of CT scans per patient increases dramatically.^6^ The increase in scans has not been shown to match an equivalent decrease in radiation per scan. Increasing the dose increases the quality of the images and leads to less diagnostic uncertainty.^7^ Multiple strategies exist to reduce the radiation dose to patients, such as choosing different examination types, decreasing the output radiation, and limiting the field exposed to radiation during the examination.

### Radiation Risks

Radiation exposure differs based on the source. Occupational exposure spreads the dose over an entire career.^8,9^ In contrast, medical imaging concentrates a high dose of radiation in short durations.^10^ The most widely accepted model for radiation exposure is the linear no-threshold model in which no amount of radiation is considered safe.^11,12^ However, this is only a model, and it overestimates the risk of sarcomas and underestimates cancers in susceptible populations.^11,13,14^ Abdominal CT scans have the highest radiation dose and cumulative dose of any imaging modality.^15^

### Methods to Reduce Risk

Software can reduce the amount of radiation exposure in multiple ways. The current to the x-ray tube can be modulated and can reduce radiation by approximately 40% for abdominal CTs without loss of quality.^16,17^ A computer can be used to create a model and reconstruct images that can reduce radiation by 34% to 42%.^18,19^ The downside is that creating a model is computationally difficult and can severely impact the turnaround time of a study.^20,21^ Instead of creating a new model for every scan, a computer can use statistical rules such as adaptive statistical iterative reconstruction (ASIR) by GE Healthcare.^22-24^ While this method is significantly faster computationally than creating a model, the diagnostic quality of the scan decreases the more these statistical rules are applied.

Based on the belief that lower-dose CT scans pose a lower risk of danger, our study purpose was to determine if the clinical application of ASIR reduced radiation dose in abdominal scans in a medium-sized healthcare system without changing ordering preferences, increasing the number of examinations requested, or increasing the CT scan coverage. Specifically, we sought to assess if the introduction of low-dose CT changed the number of examinations ordered, the number of phases requested, and the lengths of scans acquired by the technologists. In other words, we wondered if the effect of low-dose CT was similar to the consumption of low-fat foods labeled “lite” that you often end up eating more of.

## METHODS

This retrospective study was approved by the institutional review board; patient consent was not required. All data were managed in compliance with the Health Insurance Portability and Accountability Act. All examinations were performed on GE LightSpeed VCT CT scanners (GE Healthcare). ASIR was introduced at a level of 40% combined with 60% filtered back projection for the final image. Tube modulation protocols were unchanged during the study time frame.

Patient billing data for CT scans were gathered for the year 2013. The anonymized data were sorted by body parts scanned and by frequency. Examinations of abdomen and abdomen/pelvis and renal stone studies were aggregated for analysis.

A campaign for low-dose CT scans was started, and data from 2 months prior and 2 months after the initiation of the campaign were collected and analyzed using unpaired *t* tests for differences in radiation dose, overall area scanned, and number of phases requested.

In phase 1, we assessed the impact of ASIR by selecting patients who underwent a CT scan of the abdomen 1 month before and 1 month after the transition to statistical reconstruction in July and November 2014. Contrast and noncontrast studies were not differentiated. The anterior-posterior (AP) and lateral dimensions were measured on the widest segment of the abdomen in the mid portion of the scan. Radiation exposure was reported by CT scanners as a CT dose index volume (CTDI_vol_) value for every scan phase. A size-specific dose estimate (SSDE) was then calculated to better indicate the radiation dose, accounting for body habitus, and to validate the CTDI_vol_ measurements. Unpaired *t* tests were performed on the pre-ASIR and post-ASIR patient sets comparing patient demographics, AP dimensions, CTDI_vol_, and normalized SSDE values. *P*-values were determined for each. Histograms comparing dose and frequency of the CTDI_vol_ and SSDE datasets were created in Excel (Microsoft, 2015).

In phase 2, we analyzed the number of scan phases and the total radiation dose as a proxy to see if awareness of low-dose strategies had unknowingly increased the dose exposure using the same representative population during the months of July and November as in phase 1. The total dose length product (DLP) and the total number of phases for each patient encounter were recorded. Total DLP accounts for the scan length and for the larger radiation burden of an abdomen/pelvis scan vs an abdominal scan. The effect of a larger scan length on patient radiation exposure is not accounted for in the reported SSDE and CTDI_vol_ values. Because of the tube current modulation, DLP does not directly relate to the CTDI_vol_ multiplied by the length of the scan. Instead, internal calculations of the CT scanner reproduce a DLP while taking into account the varying dose along the long z-axis of the patient. For the data analysis, we relied upon the reported DLP as noted in the dose report that summed all phases of a single patient encounter. Scout scans were not recorded on the dose report for the institutional scanners.

Unpaired *t* tests were performed on the pre-ASIR and post-ASIR DLP patient sets comparing demographics, number of scans, and total DLP for each patient encounter.

During phase 3, we expanded the patient selection to include June to July for pre-ASIR and October to November for post-ASIR to allow analysis of physician ordering habits for both the number of examinations ordered and the number of phases per examination. Unpaired *t* tests were then performed using the expanded patient sets including patient demographics, number of scans, and total DLP for each patient encounter.

DLP was multiplied by the appropriate k-factor for abdominal scans in an aggregate manner to predict population-based risks for before and after implementation of ASIR.^25^ The k-factor reflects the aggregate risk of all abdominal organs and other irradiated organs during abdominal scans. DLP was then used to calculate population-based radiation risks.

## RESULTS

Analysis of the billing data showed that 31% (75,190/239,834) of the total hospital system's CT scans involved the abdomen (Table 1). Because of the large percentage, we focused on the impact of ASIR on abdominal scans. Abdominal CT scans also most closely resemble the 32 cm phantom measurements used in calculating dose exposure.

**Table 1.** Number of Computed Tomography (CT) Scans in 2013 Based on Aggregated Billing Data

Type	Number of Scans
Head	61,811
Neck	6,089
Chest	41,014
Abdomen^a^	8,601
Abdomen/pelvis^a^	65,250
Renal stone study^a^	1,339
Pelvis	1,316
Spine	13,068
Extremities	3,784
CT-guided study	1,294
CT-angiogram	20,896
Other	15,372
Total	239,834

^a^Abdomen and abdomen/pelvis scans and renal stone studies were combined for analysis (n=75,190).

### Phase 1

Phase 1 results of the unpaired *t* tests for July and November are shown in Table 2. Slightly more scans were performed in July than November (243 vs 233). The pre-ASIR and post-ASIR groups were not statistically significant when comparing age (52 ± 20 vs 53 ± 20 years, *P*=0.59), AP dimension (26.4 ± 5.3 vs 26.1 ± 5.0 cm, *P*=0.48), AP+lateral dimensions (60.8 ± 9.8 cm vs 60.3 ± 9.8 cm, *P*=0.62), or length of scan (45.1 ± 7.5 cm vs 43.7 ± 10.4 cm, *P*=0.08), respectively.

**Table 2.** Patient Demographics and Computed Tomography Factors Analyzed Before and After Iterative Reconstruction Presented by Analysis Phase

	Assessment Timepoint		
Phase/Variables	Pre-ASIR, July	Post-ASIR, November	
Phase 1	n=243	n=233	*P*-Value
Mean age, years, all patients (range)	52 ± 20 (10-92)	53 ± 20 (4-94)	0.59
Male, n (%)	112 (46)	106 (45)	
Male mean age, years (range)	53 ± 18 (10-87)	56 ± 20 (4-89)	0.45
Female, n (%)	131 (54)	127 (55)	
Female mean age, years (range)	51 ± 21 (16-92)	51 ± 21 (20-94)	1.0
Mean CTDI_vol_, mGy	17.5 ± 5.7	11.0 ± 6.0	<0.001
Mean AP dimension, cm	26.4 ± 5.3	26.1 ± 5.0	0.48
Mean lateral dimension, cm	34.4 ± 4.9	34.3 ± 5.1	0.80
Mean AP+lateral dimensions, cm	60.8 ± 9.8	60.3 ± 9.8	0.62
Mean length of scan, cm	45.1 ± 7.5	43.7 ± 10.4	0.08
Mean SSDE (AP), mGy	20.3 ± 4.6	12.5 ± 5.1	<0.001
Mean SSDE (AP+lateral), mGy	20.8 ± 5.0	12.7 ± 5.1	<0.001
Mean number of phases	1.27 ± 0.60	1.29 ± 0.63	0.79
	**Pre-ASIR, July**	**Post-ASIR, November**	
**Phase 2**	**n=199**	**n=184**	
Mean age, years, all patients (range)	52 ± 20 (10-92)	52 ± 21 (4-94)	1.0
Male, n (%)	90 (45)	84 (46)	
Male mean age, years (range)	54 ± 19 (10-87)	53 ± 21 (4-89)	0.74
Female, n (%)	109 (55)	100 (54)	
Female mean age, years (range)	50 ± 21 (16-92)	50 ± 21 (20-94)	1.0
Mean number of phases	1.3 ± 0.6	1.3 ± 0.6	0.79
Mean DLP, mGy*cm	1,076 ± 527	715 ± 512	<0.001
	**Pre-ASIR, June/July**	**Post-ASIR, October/November**	
**Phase 3**	**n=408**	**n=389**	
			
Mean age, years, all patients (range)	52 ± 20 (6-97)	50 ± 21 (4-94)	0.17
Male, n (%)	157 (38)	171 (44)	
Male mean age, years (range)	54 ± 19 (10-97)	52 ± 21 (4-93)	0.37
Female, n (%)	251 (62)	218 (56)	
Female mean age, years (range)	51 ± 21 (6-93)	49 ± 21 (7-94)	0.31
Mean number of phases	1.2 ± 0.5	1.3 ± 0.6	0.15
Mean DLP, mGy*cm	1,069 ± 634	676 ± 480	<0.001

AP, anterior-posterior; ASIR, adaptive statistical iterative reconstruction; CDTI_vol_, computed tomography dose index volume; DLP, dose length product; SSDE, size-specific dose estimate.

Analysis of the CTDI_vol_ in the pre-ASIR and post-ASIR groups showed a statistically significant reduction of 37% (17.5 ± 5.7 mGy vs 11.0 ± 6.0 mGy, respectively, *P*<0.001). After converting to SSDE using the lateral dimension, this reduction increased to 38% and was also statistically significant (20.3 ± 4.6 mGy in the pre-ASIR group vs 12.5 ± 5.1 mGy in the post-ASIR group, *P*<0.001). The tabulation for SSDE could not use both the AP+lateral dimensions because 36% of the patients prior to ASIR and 46% of the patients after ASIR extended beyond the scanner field of view. All patients had an AP dimension that was measurable, and no patients’ AP dimension extended beyond the field of view.

Histograms for CTDI_vol_ and SSDE pre-ASIR and post-ASIR by frequency are presented in the Figure. A visible shift occurred, with the post-ASIR 75th percentile of 8.0 mGy falling well below the pre-ASIR 25th percentile of 17 mGy. The leftward shift shows the impact ASIR had on all studies. The outlier in the histogram represents a virtual colonoscopy, the only one performed during the study's time frame.

**Figure.**
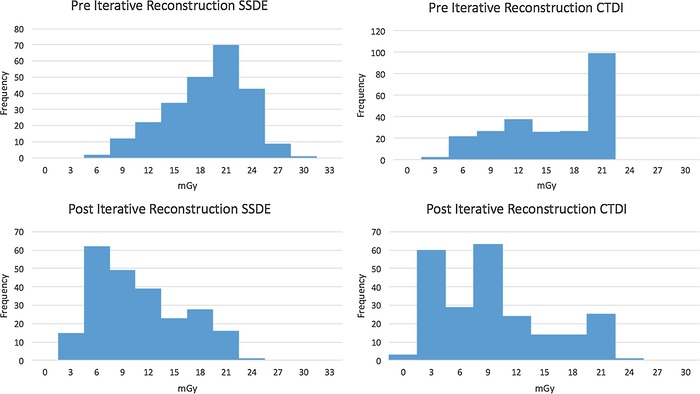
**Histograms of size-specific dose estimate (SSDE) and computed tomography dose index (CTDI) volume by frequency before and after iterative reconstruction.**

Comparison of the pre-ASIR and post-ASIR patient encounters revealed similar patterns as individual scan phases. The number of phases in the 2 groups (1.27 ± 0.60 vs 1.29 ± 0.63, *P*=0.79) was not statistically different. This finding shows that implementation of low-dose CT software did not result in an offsetting increase in the field of view selected by technologists or in an increase in physicians ordering multiphase examinations.

### Phase 2

In the phase 2 analysis (Table 2), total DLP decreased by 34% (1,076 ± 527 mGy*cm pre-ASIR vs 715 ± 512 mGy*cm post-ASIR, *P*<0.001). This reduction is similar to the reduction shown on a phase basis and extends the reduction to the entire patient encounter.

### Phase 3

For the phase 3 analysis (Table 2), the DLP data were extended to include 2 months prior to (n=408) and 2 months after (n=389) the ASIR intervention (total n=797; average age=51.0 ± 20.5 years; range, 4 to 97 years). Again, patient ages (52 ± 20 years pre-ASIR vs 50 ± 21 years post-ASIR, *P*=0.15) and the number of scan phases (1.2 ± 0.5 pre-ASIR vs 1.3 ± 0.6 post-ASIR, *P*=0.15) in the 2 groups were not statistically different. In this extended patient selection, total DLP decreased by 37% (1,069 ± 634 mGy*cm pre-ASIR vs 676 ± 480 mGy*cm post-ASIR, *P*<0.001). This finding shows that patients received less radiation overall during the examination and that this effect did not diminish during the short time period after the introduction of ASIR.

## DISCUSSION

This study sought only to compare the impact of software in CT scanners that were already installed and physician ordering preferences. Only ASIR 40% with filtered back projection at 60% was included because this level was found to be diagnostically acceptable in the literature reviewed.^20^ Abdominal CT scans were the focus of this study because of the similarity to the 32 cm phantom, the overall number of abdominal CT scans in the patient population, and the larger amount of radiation required for abdominal CT scans compared with other CT scans.

Based on our analysis of abdominal CT scans, employing a dose-reduction strategy that reduces the individual phase radiation by ASIR or other means will decrease the total amount of radiation to the population without an increase in the number of scans or increased ordering of more radiation-intense examinations by physicians. This reduction translates to a cumulative reduction of 433,000 mSv in the course of the year. Based on conservative population studies correlating radiation exposure to malignancies in the population from atomic bomb exposure, a yearly reduction of 433,000 mSv from abdominal CT scans could reduce the number of malignancies possibly attributed to medical radiation by 22.2 per year (based on the extrapolation of 1 malignancy per 2,000 mSv of exposure to the whole body on a population level).^4^

Radiation exposure thresholds are changing as lower-dose imaging becomes more prevalent. Although pediatric studies attribute a proximal cause of some types of cancer to radiation exposure, adult incidents of cancer may have a longer time to onset compared to pediatric patients.^6,7^ Lower doses may in turn relate to a longer time to onset of malignancy. All models of radiation exposure support a reduction in current medical doses to as low as reasonably acceptable.

The reduction secondary to ASIR in this hospital study using the SSDE methodology was in close agreement with the reduction of 37% using CTDI_vol_ without accounting for patient size. More important, the distribution of radiation dose shifted lower without the need to replace existing CT scanners.

Further reductions could be achieved by shifting clinician ordering habits away from unnecessary multiphase examinations. An examination both without and with contrast will have twice the radiation of a scan with contrast, regardless of the dose-saving strategies deployed. Based on the data provided, more than 60% of examinations have at least 2 phases, with most of them being noncontrast phases requested by ordering clinicians.

The only limitations in our sampling came from incorrect CTDI_vol_ calculations based on the 16 cm phantom instead of the 32 cm phantom. This situation only occurred with 2 patient scans. Twelve children were excluded from this study of adult patients. Our patients had differing abdominal lateral dimensions, and 41% extended beyond the scanner field of view, thanks to a hearty American diet. Our sample size would have been severely decreased if we had used the AP+lateral methodology recommended by the American Association of Physicists in Medicine.^26^ To compensate, we made recommendations to normalize radiation exposure based on patient size, with theoretical models showing how radiation exposure would be reduced for larger patients based on their SSDE. Further, different disease processes such as ascites bring about a larger abdominal girth without an increase in body fat that would predictably attenuate the exposure to radiation. This confounder was overcome by a large sample size and consistency in AP measurements.

## CONCLUSION

Implementation of software reduction strategies on existing CT scanners can reduce radiation exposure by approximately 37% and shifts the distribution of radiation scans into a more favorable profile without changing physician ordering preferences because of the perceived lower dose. While pediatric studies have been the primary focus of radiation control, abdominal CT scans have the highest radiation burden and are the most common type of CT scans as shown by our data. Extrapolating from our abdominal CT scan data, employing a dose-reduction strategy that reduces the individual phase radiation by ASIR or other means can decrease the total amount of radiation to the population without an increase in the number of scans or increased ordering of more radiation-intense examinations by physicians.

## Notes

This article meets the Accreditation Council for Graduate Medical Education and the American Board of Medical Specialties Maintenance of Certification competencies for Patient Care and Medical Knowledge.
